# Kinetic Characterization and Inhibitor Screening of Pyruvate Kinase I From *Babesia microti*

**DOI:** 10.3389/fmicb.2021.710678

**Published:** 2021-09-16

**Authors:** Xiaomeng An, Long Yu, Sen Wang, Yangsiqi Ao, Xueyan Zhan, Qin Liu, Yangnan Zhao, Muxiao Li, Xiang Shu, Fangjie Li, Lan He, Junlong Zhao

**Affiliations:** ^1^State Key Laboratory of Agricultural Microbiology, College of Veterinary Medicine, Huazhong Agricultural University, Wuhan, China; ^2^Key Laboratory of Animal Epidemical Disease and Infectious Zoonoses, Ministry of Agriculture, Huazhong Agricultural University, Wuhan, China; ^3^Key Laboratory of Preventive Veterinary Medicine in Hubei Province, Wuhan, China

**Keywords:** *Babesia microti*, pyruvate kinase I, inhibitors, kinetic characterization, inhibition assay

## Abstract

The apicomplexan *Babesia microti* is a main pathogenic parasite causing human babesiosis, which is one of the most widely distributed tick-borne diseases in humans. Pyruvate kinase (PYK) plays a central metabolic regulatory role in most living organisms and catalyzes the essentially irreversible step in glycolysis that converts phosphoenolpyruvate (PEP) to pyruvate. Hence, PYK is recognized as an attractive therapeutic target in cancer and human pathogens such as apicomplexans. In this study, we cloned, expressed, and purified *B. microti* PYK I (BmPYKI). Western blotting illustrated that anti-rBmPYKI antibody could specifically recognize the native BmPYKI protein in the lysate of *B. microti* with a 54-kDa band, which is consistent with the predicted size. In addition, the enzymatic activity of the purified recombinant PYKI (rPYKI) was tested under a range of pH values. The results showed that the maximum catalytic activity could be achieved at pH 7.0. The saturation curves for substrates demonstrated that the *K*_m_ value for PEP was 0.655 ± 0.117 mM and that for ADP was 0.388 ± 0.087 mM. We further investigated the effect of 13 compounds on rBmPYKI. Kinetic analysis indicated that six inhibitors (tannic acid, shikonin, apigenin, PKM2 inhibitor, rosiglitazone, and pioglitazone) could significantly inhibit the catalytic activity of PYKI, among which tannic acid is the most efficient inhibitor with an IC_50_ value 0.49 μM. Besides, four inhibitors (tannic acid, apigenin, shikonin, and PKM2 inhibitor) could significantly decrease the growth of *in vitro*-cultured *B. microti* with IC_50_ values of 0.77, 2.10, 1.73, and 1.15 μM. Overall, the present study provides a theoretical basis for the design and development of new anti-*Babesia* drugs.

## Introduction

*Babesia microti* is an intraerythrocytic apicomplexan protozoan distributed worldwide and causes an emerging global zoonotic disease associated with human babesiosis. It is mainly transmitted via bites of the tick vector, *Ixodes scapularis* (Gray et al., 2010; Vannier and Krause, 2012). However, blood transfusion has become one of the may pathways for the infection of babesiosis in the United States, and the mortality rate of transfusion-transmitted babesiosis has been estimated to be 20%, which poses a serious threat to the supply of safe blood (Levin and Krause, 2016; Moritz et al., 2016). Currently, there are no effective vaccines for humans against *B. microti*, and the combination therapy is generally recommended for the clinical treatment of babesiosis, including azithromycin and atovaquone for mild cases, and clindamycin combined with quinine for severe cases (Krause et al., 2000; Sanchez et al., 2016). Although these combined therapies are effective in controlling infection, numerous reports have demonstrated that the negative side effects during the treatment, such as hearing loss and tinnitus, which largely limit their clinical application (Clyde et al., 1975; Wormser et al., 2010). Atovaquone or azithromycin can block the mitochondrial electron transport pathway in *B. microti* to inhibit its growth. However, *B. microti* can rapidly develop resistance to atovaquone or azithromycin (Fry and Pudney, 1992), leading to treatment failure and relapsing infection. Due to the emergence of antimicrobial resistance, therapeutic regime is usually adjusted to increase the dose of drugs or use different combined treatments, such as doxycycline and proguanil, which may inhibit the apicoplast of *B. microti* (Wormser et al., 2010; Iguchi et al., 2014), or adopt various other pharmacologic interventions such as the addition of pentamidine, chloroquine, quinine, and mefloquine with variable effects (Brasseur et al., 1998), which can suppress DNA synthesis. Therefore, the exploration of new drug targets is essential for the treatment of babesiosis in the future.

*Babesia microti* highly relies on glucose fermentation for energy production and redox regulation (Cornillot et al., 2012), indicating that glycolysis is a promising target for new drugs against babesiosis. Pyruvate kinase (PYK) catalyzes the essentially irreversible transphosphorylation of ADP at the expense of phosphoenolpyruvate (PEP), producing pyruvate and ATP, which plays an important regulatory role in glycolysis (Ernest et al., 1998; Maeda et al., 2003). Furthermore, the consumption of glucose of the red blood cells (RBCs) infected by *Plasmodium falciparum* was 100-fold higher than that of uninfected RBCs; and correspondingly, the content of PYK in *P. falciparum* showed a dramatic increase, indicating that PYK can serve as a potential target of drugs (Roth et al., 1988). However, the conserved structure of the active site of PYK increases the difficulty in developing selective inhibitors against the enzyme. Almost all PYKs are allosterically regulated by various physiological allosteric modulators (Muñoz and Ponce, 2003; Morgan et al., 2010). In order to regulate the glycolysis flux in response to increase in the PEP level, almost all tetrameric PYKs could be activated by the substrate PEP. ATP is highly specific for the allosteric inhibition of PYK L (Carbonell et al., 1973). In addition, PYK is also regulated by anisotropic effectors. For example, fructose-1,6-bisphosphate (FBP) is the most widely known allosteric activator of the enzyme in bacteria, yeast, and mammals (Jurica et al., 1998); and fructose-2,6-bisphosphate is the primary allosteric effector in trypanosomatids (Callens et al., 1991). Therefore, the differences in the allosteric mechanism of PYK offer a unique opportunity for selective targeting of the enzyme.

There are four mammalian PYK isoforms (PKL, PKR, PKM1, and PKM2), which are distributed in different tissues. PKL and PKR are the major isoforms in the liver and RBCs, respectively, both of which are allosterically regulated by FBP. PKM1 is a highly active non-allosteric form in tissues that rapidly produce ATP in large amounts, such as the heart, brain, and skeletal muscle. Phenylalanine has an allosteric inhibitory effect on PKM1 in the muscle and brain (Feksa et al., 2003). PKM2 is expressed in all proliferating cells, leukocyte, lung, spleen, kidney, and adipose tissues. Tumors caused by various tissue types are highly dependent on glycolysis for energy metabolism. Hence, PKM2 is generally expressed in tumor cells.

Accordingly, screening of specific inhibitors of PKM2 targeting the metabolism of cancer cells can contribute to the development of new drugs for cancer treatment. In some recorded experiments, shikonin suppressed PKM2-mediated aerobic metabolism by preventing the tetramer-to-dimer conformational switch of PKM2 to inhibit tumor aerobic glycolysis and tumor growth. Besides, shikonin could reduce the phosphorylation of PKM2 in tumor cells, though it did not reduce the total cellular PKM2 level (Zhao et al., 2018). The inhibition of PKM2 activity by lapachol in melanoma cells brought about a decline of ATP level and inhibition of cell proliferation due to a high-affinity binding pocket for lapachol in the PKM2 structure (Shankar Babu et al., 2018). Tannic acid, a natural polyphenolic acid primarily existing in grape and green tea, has strong antioxidant and anticancer properties. Tannic acid directly binds to PKM2 lysine residue 433, a selective druggable site, which leads to the dissociation of PKM2 tetramer. Notably, it merely blocks the metabolic activity of PKM2 rather than that of PKM1, making it a promising PKM2 inhibitor for the prevention of colorectal cancer (CRC) (Yang et al., 2018). Previous studies have revealed that flavone and its analogs could effectively inhibit PKM2 activity, including apigenin, wogonin, 3-hydroxyflavone, 5-hydroxyflavone, 6-hydroxyflavone, and 7-hydroxyflavone (Aslan and Adem, 2015; Shan et al., 2017). Furthermore, *Carpesium abrotanoides* L. (PCA) downregulates the expression of PKM2 and leads to its cellular translocation (Chai et al., 2019), and benserazide directly binds to and diminishes PKM2 activity to suppress the growth of melanoma cells (Zhou et al., 2020).

Currently, there has been limited research on PYK inhibitor in the protozoan parasite. Ethyl pyruvate can effectively kill trypanosomes within 3 h of exposure through net ATP depletion by inhibiting PYK (Worku et al., 2015). In addition, the complex crystal structure of PYK from *Leishmania mexicana* or *Trypanosoma cruzi* demonstrated that suramin and other three dyes (Ponceau S, acid blue 80, and benzothiazole-2,5-disulfonic acid) were bound at its ADP/ATP active sites (Morgan et al., 2011). In the design of PYK inhibitors for *Trypanosoma* glycolysis, furosemide showed an inhibitory effect on PYK and killed trypanosomes at a low concentration (Nowicki et al., 2008).

In this study, we report the cloning, prokaryotic expression, immunogenicity, and detailed kinetic properties of *B. microti* PYK I (BmPYKI). Additionally, we demonstrate that tannic acid, apigenin, shikonin, and PKM2 inhibitor can inhibit the activity of rBmPYKI and suppress the growth of *B. microti*. However, further research is needed to evaluate the inhibition mechanisms of these inhibitors on BmPYKI and to design and develop more efficient compounds.

## Materials and Methods

### Ethics Statement

The experimental animals were housed and treated in accordance with the stipulated rules for the Regulation of the Administration of Affairs Concerning Experimental Animals of China. All experiments were performed under the approval of the Laboratory Animals Research Centre of Hubei Province and Huazhong Agricultural University (permit number: HZAUMO-2019-052).

### Parasites and Animals

*Babesia microti* strain ATCC^®^ PRA-99^TM^ was provided by the National Institute of Parasitic Diseases, Chinese Center for Disease Control and Prevention (Shanghai, China), and maintained in our laboratory. BALB/c mice were administered with 1 × 10^7^ parasites (100 μl of infected blood) by intraperitoneal injection. Parasites were collected from infected BALB/c mice when parasitemia reached 30%. All mice and Japanese rabbits were purchased from the Animal Center of Huazhong Agricultural University.

### *Babesia microti* Pyruvate Kinase I Cloning, Recombinant Expression, and Purification

The gene fragment BmPYKI (GenBank no. XM_012794432.2) was amplified from the cDNA of *B. microti* by using two primers BmPYKI-F (5′-GCGGATCCATGAA-TAGAATAATCAAAG-3′) and BmPYKI-R (5′-GCGTCGACTTAAACATTAA-GAATTTT AAGG-3′), which contained the restriction sites of *Bam*HI and *Sal*I (underlined) (Takara, Beijing, China), respectively. Then the expression plasmid (pGEX-6p-1-BmPyKI) was constructed and confirmed by restriction enzyme digestion and sequencing.

The constructed plasmid was transformed into *Escherichia coli* BL21. *E. coli* was cultured at 37°C and subsequently induced overnight with 1 mM of isopropyl-β-D-thiogalactopyranoside (IPTG; Biosharp, Anhui, China) at 28°C. Cells were harvested by centrifugation, and the pellets were re-suspended in moderate binding buffer (1× phosphate-buffered saline (PBS), pH 7.4); after centrifugation at 500 × *g* for 10 min at 4°C, the supernatant was purified by GST affinity column according to the manufacturer’s instructions (GE Healthcare, Waukesha, WI, United States). 3C protease was used to cut the GST tag from the purified protein. The purified protein was added to GST affinity column with 10 ml of PBS, 20 μl of DTT (1 mM), and 300 μl of 3C protease (1 mg/ml), which was used to cut the fusion GST, and then incubated at 4°C overnight. The GST tag was eluted from GST affinity column by 10 ml of elution buffer. rBmPYKI without GST tag was separated from the GST affinity column by filtration. Supernatant containing purified rBmPYKI was placed in a dialysis bag and slowly concentrated with sucrose. Protein concentration was determined using a bicinchoninic acid (BCA) protein assay kit (Beyotime Biotechnology, Shanghai, China). Finally, the purified protein was stored at −80°C until use.

### Preparation and Purification of Polyclonal Antibody Against BmPYKI

The anti-rBmPYKI was produced with purified rBmPYKI–GST fusion protein. Briefly, rBmPYKI–GST was mixed with an equal volume of Freund’s complete adjuvant (MilliporeSigma, Burlington, MA, United States) at a final concentration of 500 μg/ml. Subsequently, 1 mg of rBmPYKI–GST was subcutaneously injected into the back of two Japanese Long Ear Rabbits (4 months old, about 2.5 kg). The rabbits were immunized again with 500 μg of rBmPYKI–GST in Freund’s incomplete adjuvant (MilliporeSigma) every 2 weeks until the fourth immunization.

Blood was collected 7 days after the last immunization, placed at room temperature for 1 h, and then incubated at 4°C overnight. The supernatant (antisera) was collected via centrifugation at 500 × *g* for 10 min. Anti-rBmPYKI serum was purified by Protein A Berpharose FF following the manufacturer’s instructions (GE Healthcare, Waukesha, WI, United States).

### Western Blotting Analysis

To determine native BmPYKI in *B. microti*, Western blotting was performed. Briefly, the lysates of *B. microti* were separated by sodium dodecyl sulfate–polyacrylamide gel electrophoresis (SDS–PAGE, containing 12% acrylamide) and transferred to polyvinylidene difluoride (PVDF) membrane. The membranes were blocked with Tris-buffered saline Tween-20 (TBST) containing 1% (w/v) bovine serum albumin (BSA; BioFroxx, Hesse, Germany) for 1 h at room temperature. The membranes were incubated with rabbit polyclonal anti-BmPYKI serum (1:100 dilution) for 2 h at room temperature, with pre-immune serum as the negative control. Purified rBmPYKI was used to react with the rabbit polyclonal antibody as a positive control. Then the membranes were soaked in peroxidase-conjugated goat anti-rabbit immunoglobulin G (1:5,000 dilution) for 1 h at room temperature. The reactions were detected by WesternBright^TM^ ECL (Advansta, San Jose, CA, United States).

### Enzyme Kinetics and Inhibition Assays

To determine the enzymatic activity of purified rBmPYKI, the GST tag was removed from the recombinant protein; and the indirect coupled lactate dehydrogenase (LDH) assay was applied. Recombinant *B. microti* LDH (rBmLDH) protein, the only one isoform in *B. microti*, was expressed and purified according to previously published protocol (Yu et al., 2019). The standard enzyme kinetic assay was conducted by monitoring the decrease in NADH at a wavelength of 340 nm using a microplate reader (BioTek, Winooski, VT, United States). One unit of the PYK activity here was defined as 1 μmol of NADH oxidized per minute at 37°C. The samples were continuously measured at 2, 7, 12, 17, 22, and 27 min. To determine the effects of pH and the concentration of Mg^2+^ and K^+^ on rBmPYKI, the assay was performed in the pH range of 5.5–8.5, and the Mg^2+^ and K^+^ concentrations ranged from 0 to 200 mM (0, 25, 50, 75, 100, 125, 150, 175, and 200 mM). The standard assay mixture (200 μl) contained 50 mM of Tris-HCl (pH 7.0), 50 mM of MgCl_2_, 20 mM of KCl, 0.5 mM of NADH (MilliporeSigma), 3 mM of PEP (MilliporeSigma), 1 mM of ADP (MilliporeSigma), 100 ng of rBmLDH (20 U), and 100 ng of rBmPYKI. The initial velocity for the rate of reduction of NADH in the first 5 min was calculated, and the relative percentage activity was determined. The kinetic parameters for PEP (at 0.5, 1, 2, 3, 4, and 5 mM) under saturating ADP conditions (3 mM) were determined. The Michaelis–Menten constant (*K*_m_) for ADP under 7 mM of PEP (saturation) was determined at 0.05, 0.1, 0.2, 0.4, 0.6, 0.8, 1.0, 1.5, and 2.0 mM.

The activity of rBmPYKI was also detected by using Pyruvate Kinase Assay Kit (MilliporeSigma) according to the manufacturer’s instruction, which provided a simple and direct procedure for measuring pyruvate concentration by coupling fluorescent peroxidase. PYK activity was calculated as nmol/min/ml = milliunit/ml, which means that one milliunit (mU) of PYK is defined as the amount of enzyme that will transfer a phosphate group from PEP to ADP to generate 1.0 nmol of pyruvate per minute at 25°C.

A total of 13 small molecular compounds including apigenin, tannic acid, shikonin, PKM2 inhibitor, pioglitazone, rosiglitazone, wogonin, suramin, disodium monofluorophosphate, lapachol, flavone, and 6-hydroxyflavone (TargerMol, Shanghai, China), were prepared in dimethyl sulfoxide (DMSO) according to the manufacturer’s instructions. Indirect coupled LDH assay was performed to determine the effects of the 13 compounds on rBmPYKI activity. These compounds were five-fold serially diluted to the concentrations of 1.953125 mM, 390.625 μM, 78.125 μM, 15.625 μM, 3.125 μM, 625 nM, 125 nM, 25 nM, 5 nM, and 1 nM and then used to calculate the half maximal inhibitory concentration (IC_50_) value of rBmPyKI. Then, the rBmPYKI activity after incubation with the 13 compounds was detected by using Pyruvate Kinase Assay Kit. The inhibition rate was calculated by using the following equation:


Inhibition(%)=(ΔA-controlΔAsample)/ΔAcontrol×100%.

### Growth Inhibition and Rescue Assay of *Babesia microti* by *in vitro* Culture

Infected mouse blood was collected and centrifuged at 845 × *g* for 5 min, and the infected mouse RBCs was isolated. The RBCs infected by *B. microti* were diluted to 5% with uninfected mouse and human RBCs (10:1). The parasites were cultured in a total volume of 100 μl of HL-1 medium containing glucose and L-glutamine (Gibco, Beijing, China) with 20% bovine serum in a 96-well plate and treated with different concentrations of the small molecular compounds: apigenin and PKM2 inhibitor, 5 nM, 50 nM, 500 nM, 5 μM, 50 μM, and 500 μM; tannic acid and shikonin: 32 nM, 160 nM, 800 nM, 4 μM, 20 μM, and 100 μM. The parasites were incubated at 5% CO_2_, 37°C for 72 h. Drug-free wells were set up as blank control. All treatments were carried out in triplicate. After 72 h of incubation, parasitemia was examined by Giemsa staining under a microscope.

In the rescue assay, 2 mM of pyruvic acid or 2 mM of ATP (MilliporeSigma) were added into the medium containing 1 μM of tannic acid (70% inhibition). About 10 μM of diminazene aceturate (DA; MilliporeSigma) was used as the positive control, and DMSO was used as the negative control because tannic acid was diluted in DMSO. The group only containing medium without any treatment was used as the blank control. Each test was performed in triplicate.

### Cytotoxicity Assays

To estimate the cellular toxicity of the selected compounds, including apigenin, tannic acid, PKM2 inhibitor, and shikonin, a growth inhibitory assay was carried out by using Vero and HFF cells treated with different concentrations of the compounds (Vero cells: 0.032, 0.16, 0.8, 4, 20, and 100 μM; HFF cells: 0.05, 0.5, 5, 25, 50, and 100 μM). Cell growth was measured by using Cell Counting Kit-8 (Biosharp, Beijing, China) following the manufacturer’s instruction. The cells treated with adriamycin (MilliporeSigma, 4 μM) and DMSO were set as the positive control and negative control, respectively.

### Statistical Analysis

Statistical analysis was conducted with GraphPad Prism 7.0 (GraphPad Software, La Jolla, CA, United States). Statistical significance was evaluated by one-way ANOVA; and all data were presented as the mean ± standard deviation (SD) from at least three independent experiments performed in duplicate. A value of *p* < 0.05 was considered as significant.

## Results

### cDNA Cloning, Sequence Analysis, and Purification of BmPYKI

**FIGURE 1:**
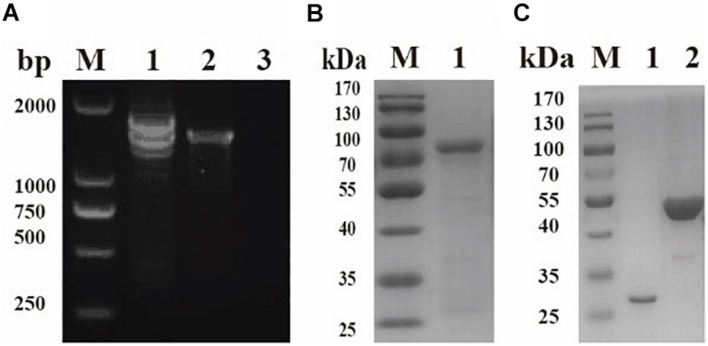
Cloning and purification of recombinant *Babesia microti* pyruvate kinase I (BmPYKI). **(A)** PCR amplification for target genes from gDNA and cDNA of *B. microti*. Lane M, DNA marker; lane 1, gDNA of *B. microti*; lane 2, cDNA of *B. microti*; lane 3, negative control. **(B)** Sodium dodecyl sulfate–polyacrylamide gel electrophoresis (SDS–PAGE) analysis of purified rBmPYKI–GST. Lane M, protein molecular marker; lane 1, purified rBmPYKI–GST protein. **(C)** SDS–PAGE analysis of purified rBmPYKI after cleavage. Lane M, protein molecular marker; lane 1, GST tag; lane 2, purified rBmPYKI without GST tag.

**FIGURE 2:**
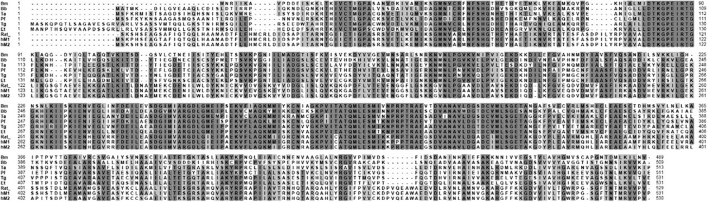
Amino acid sequence alignment of *B. microti* pyruvate kinase I (PYKI) with pyruvate kinases from other species. Accession numbers are as follows: Bm, *B. microti* (XP_012649886); Bb, *Babesia bovis* (XP_001611829); Ta, *Theileria annulata* (XP_953251); Pf, *Plasmodium falciparum* (XP_966251); Tg, *Toxoplasma gondii* (XP_002364923); Et, *Eimeria tenella* (XP_013232798); Rat, rat muscle pyruvate kinase (15987970); hM1, human muscle isozyme 1 (S64635); and hM2, human muscle isozyme 2 (O18919).

The sequence of BmPYKI was obtained from National Center for Biotechnology Information (NCBI) (GenBank accession no. XP_012649886_2), and the gene was amplified from *B. microti* gDNA and cDNA (Figure 1A). The results showed that BmPYKI has a full length of 1,740 bp and contains two introns, one from position 122 to position 170 (49 bp) and the other from position 888 to position 1109 (221 bp). The open reading frame (ORF) of BmPYKI is 1,470 bp. The gene was amplified and then cloned into the pGEX-6p-1 vector. Recombinant BmPYKI (rBmPYKI) protein tag was fusion expressed with a GST tag in *E. coli* BL21 (DE3), and rBmPYKI–GST was purified as nearly 80 kDa (predicted as 79.79 kDa, Figure 1B). Then, the GST tag of rBmPYKI was cut and removed by 3C protease, resulting in a length of 54 kDa (Figure 1C). Blast analysis demonstrated that BmPYKI shares certain identities with PYKI of apicomplexan parasites, including *Toxoplasma gondii* (54.90%), *Theileria annulata* (50.20%), *P. falciparum* (52.93%), and *Eimeria tenella* (56.12%). Multiple alignments based on amino acid sequences showed that BmPYKI also shares similarities to human (43.64%) and rabbit (43.23%) PYKI (Figure 2).

### Identification of Native BmPYKI

**FIGURE 3:**
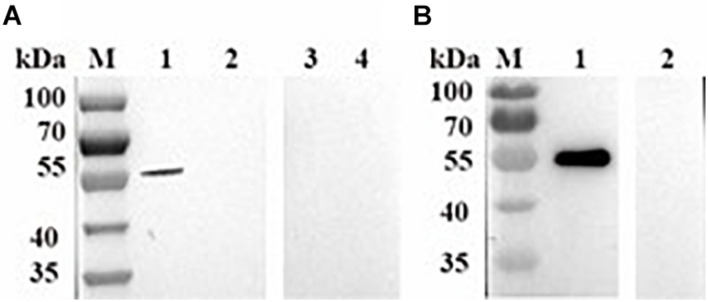
Western blotting analysis of *B. microti* pyruvate kinase I (BmPYKI). **(A)** Western blotting analysis of native PYKI in *B. microti*. Lane M, protein marker; lane 1, lysates of *B. microti* reacted with BmPYKI rabbit polyclonal antibody; lane 2, lysates of normal mouse RBCs reacted with BmPYKI rabbit polyclonal antibody; lane 3, lysates of *B. microti* reacted with serum from normal rabbit; lane 4, lysates of normal mouse RBCs reacted with serum from normal rabbit. **(B)** Western blotting analysis of rBmPYKI. Lane M, protein marker; lane 1, purified rBmPYKI reacted with BmPYKI rabbit polyclonal antibody; lane 2, purified rBmPYKI reacted with serum from normal rabbit.

To identify native BmPYKI, the lysates of *B. microti* were incubated with rabbit polyclonal anti-BmPYKI serum and pre-immune rabbit serum. Western blotting illustrated a remarkable 54-kDa band for the reaction of *B. microti* lysates with anti-BmPYKI serum. The size was consistent with the predicted size of native BmPYKI. No signal was observed from pre-immune serum and lysate of RBCs, which were used as negative controls (Figure 3A). Western blotting showed a positive band consistent with the expected band (54 kDa) in the reaction of purified rBmPYKI with anti-BmPYKI serum and pre-immune rabbit serum, and no band appeared in negative control group (Figure 3B).

### Kinetic Characteristics of rBmPYKI

**FIGURE 4:**
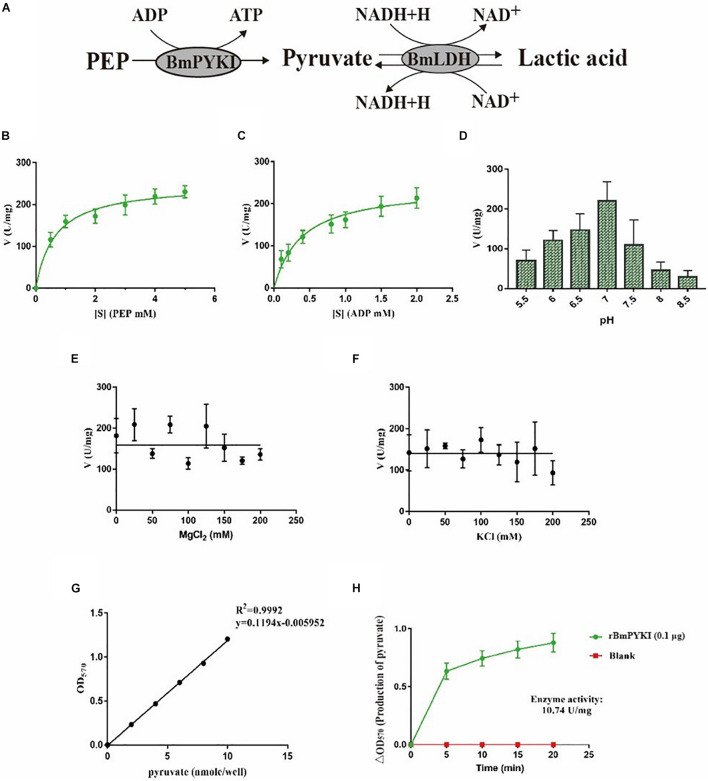
Recombinant *B. microti* pyruvate kinase I (BmPYKI) enzymatic activity and influencing factors. **(A)** Reaction scheme of indirect coupled rBmLDH assay. **(B,C)** Variations of rBmPYKI activity with increasing concentrations of phosphoenolpyruvate (PEP) and ADP. **(D)** Catalytic activity of rBmPYKI under different pH conditions. **(E,F)** Catalytic activity of rBmPYKI under different Mg^2+^ and K^+^ concentrations (0–200 mM). **(G,H)** Pyruvate standard curve and enzyme activity assay. Pyruvate standards of 0 (blank), 2, 4, 6, 8, and 10 nmol per well were specifically detected by colorimetric assay. The activity of rBmPYKI was detected at 2, 7, 12, 17, and 20 min of reaction at OD_570_ (background removed). Means ± SD (*n* = 3) from one representative of at least three independent experiments.

To characterize enzymatic activity of purified rBmPYKI, the GST tag was cut and removed by 3C protease (Figure 1C). Since the concentrations of pyruvate and ATP could not be directly measured, the indirect coupled LDH (rBmLDH) assay was employed to determine the activity of rBmPYKI by measuring the amount of NADH. A standard enzyme kinetics assay was conducted by monitoring the decrease of NADH at a wavelength of 340 nm using a microplate reader (Figure 4A). The results indicated that rBmPYKI can catalyze the reaction (the *K*_m_ value was 0.655 mM for PEP and 0.388 mM for ADP) (Figures 4B,C and Table 1). To explore the effect of pH and Mg^2+^ and K^+^ concentrations on rBmPYKI, the enzyme activity was measured in a pH range of 5.5–8.5 and increasing concentrations of Mg^2+^ and K^+^. The results revealed that the enzyme activity was the optimal at pH 7.0, while Mg^2+^ and K^+^ concentrations had no effect on rBmPYKI activity (Figures 4D–F). The optimal enzymatic conditions were 3 mM of PEP, 1 mM of ADP, and pH 7.0. Furthermore, the rate of transferring a phosphate group from PEP to ADP by rBmPYKI was further characterized by measuring the production rate of pyruvate over time, and as a result, the catalysis rate of rBmPYKI was determined to be 10.74 U/mg (Figures 4G,H).

**TABLE 1:** Kinetic parameters of rBmPYKI on different substrates (PEP and ADP).

Parameters	PEP	ADP
*K*_M_ (mM)	0.655 ± 0.117	0.388 ± 0.087
*V*_max_ (U/mg)	250 ± 10	242 ± 16
*K*_cat_ (S^–1^) *K*_cat_*K*_M_ (S^–1^⋅M^–1^)	22.5 3.44 × 10^4^	21.8 5.62 × 10^4^

*rBmPYKI, recombinant Babesia microti pyruvate kinase I; PEP, phosphoenolpyruvate.*

### Inhibition of Purified rBmPYKI

**FIGURE 5:**
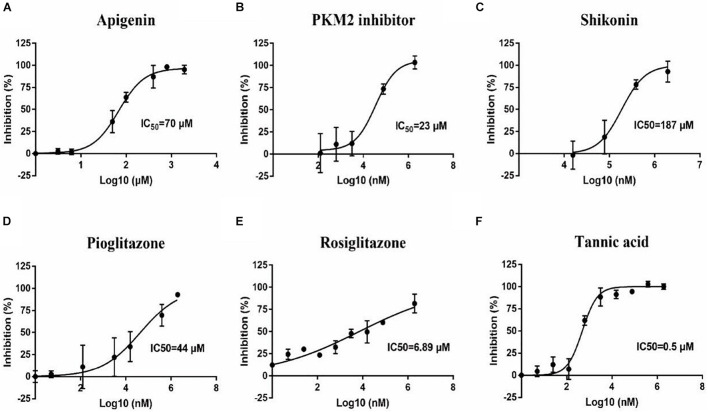
Inhibition of *B. microti* pyruvate kinase I (rBmPYKI) activity with increasing concentrations of small molecular compounds. **(A–F)** IC_50_ values of different small molecular compounds on the inhibition of rBmPYKI activity, including apigenin, shikonin, PKM2 inhibitor, rosiglitazone, and pioglitazone. The inhibition concentration range of small molecular compounds is 0–2 mM. The NADH absorbance (nmol) was measured at 2, 7, 12, 17, 22, and 27 min of reaction at OD_340_ (background removed). Standard deviations from at least three independent experiments are shown.

To confirm the effect of 13 small molecular compounds on rBmPYKI, the enzyme activity was measured with indirect coupled LDH assay (Figure 5A). The results showed that four compounds, apigenin, shikonin, PKM2 inhibitor, and pioglitazone, could significantly inhibit the activity of rBmPyKI and did not affect the LDH reaction activity (Figures 5A–D and Table 2). Besides, tannic acid and rosiglitazone could inhibit rBmPYKI enzymatic activity at a low concentration of 0.49 and 6.89 μM, respectively. There were obvious differences in the IC_50_ values against rBmPYKI and rBmLDH (Figures 5E,F and Table 2). The data illustrated that 6-hydroxyflavone, suramin, wogonin, and polydatin had an obvious inhibitory effect on rBmLDH; while disodium monofluorophosphate, lapachol, and flavone had no effect on the activity of rBmPYKI and rBmLDH (Table 2). In addition, we also investigated the effect of these compounds by using Pyruvate Kinase Assay Kit, and the results were consistent with those obtained by the indirect coupled method. Overall, apigenin, shikonin, PKM2 inhibitor, pioglitazone, rosiglitazone, and tannic acid could significantly reduce the activity of rBmPYKI.

**TABLE 2:** IC_50_ values of different small molecular compounds on the inhibition of rBmPYKI and rBmLDH.

Compounds	IC_50_	
	rBmPYKI (μM)	rBmLDH (μM)
Apigenin	70	–
Shikonin	187	–
PKM2 inhibitor	23	–
Pioglitazone	44	–
Rosiglitazone	6.89	1.86 × 10^5^
Tannic acid	0.49	23.62
6-Hydroxyflavone	389.63	827.71
Suramin	124.69	44.22
Wogonin	184.81	142.93
Polydatin	92.94	141.50
Disodium monofluorophosphate	–	–
Lapachol	2.54 × 10^5^	721.37
Flavone	–	–

*rBmPYKI, recombinant *Babesia microti* pyruvate kinase I; rBmLDH, recombinant *Babesia microti* lactate dehydrogenase.*

### Growth Inhibition of *Babesia microti in vitro*

**FIGURE 6:**
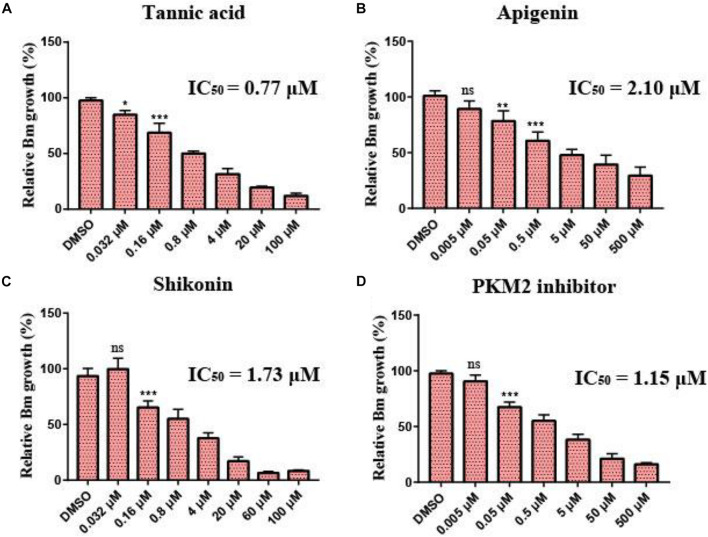
Growth inhibition of *Babesia microti* by different drugs *in vitro*. **(A–D)** *In vitro* growth rate of *B. microti* in the absence (control) or presence of different concentrations of tannic acid, apigenin, shikonin, and PKM2 inhibitor. Asterisks (*) indicate significant differences between the drug groups and the control group (* represents *p* < 0.05, ** represents *p* < 0.01, and *** represents *p* < 0.001); ns, no significance. The error bar represents mean ± SD (*n* = 3), and all charts were produced using GraphPad Prism 7.0.

To investigate the inhibitory effect of the selected small molecular compounds on *B. microti in vitro*, *B. microti*-infected RBCs were incubated with vehicle or increasing concentrations of drugs for 72 h. The 80% inhibition rate of both tannic acid and shikonin could be achieved at the concentration of 20 μM, and their IC_50_ values were 0.77 and 1.73 μM, respectively. Slight hemolysis of RBCs was observed at a high concentration of 100 μM of shikonin. The 80% inhibition rate of PKM2 inhibitor was achieved at the concentration of 50 μM, with an IC_50_ value of 1.15 μM. Apigenin exhibited a lower inhibitory effect on the growth of *B. microti* (IC_50_ = 2.1 μM) (Figures 6A–D and Table 3), and pioglitazone and rosiglitazone had no significant effect.

**TABLE 3:** *In vitro* evaluation of four compounds against the catalytic activity of rBmPYKI, asexual blood stage of *Babesia microti*, cytotoxicity on Vero, HFF cells, and selectivity index (SI).

Compounds	IC_50_ (μM)		CC_50_ (μM)		SI	
	rBmPYKI	*B. microti*	Vero	HFF	Vero	HFF
Tannic acid	0.49	0.7 ± 10.13	52.97 ± 2.80	36.46 ± 4.75	74.61	51.35
Shikonin	187	1.73 ± 0.69	2.37 ± 0.26	0.63 ± 0.40	1.37	–
Apigenin	70	2.10 ± 1.16	58.56 ± 2.97	32.97 ± 1.18	27.88	15.70
PKM2 inhibitor	23	1.15 ± 0.11	39.21 ± 5.05	18.59 ± 2.46	34.10	16.16

*rBmPYKI, recombinant *B. microti* pyruvate kinase I.*

To calculate the selectivity index (SI) of the four compounds (tannic acid, shikonin, PKM2 inhibitor, and apigenin) screened above, the cytotoxicity was calculated by using Cell Counting Kit-8 to detect the growth of Vero and HFF cells. The results indicated that the SI of tannic acid was 74.61 and 51.35 on Vero and HFF cells, respectively (Table 3), suggesting that it can selectively inhibit the growth of *B. microti* with a low cytotoxicity. However, shikonin showed a much lower SI (SI = 1.37) than tannic acid, suggesting that it may have a high toxicity on mammalian cells. PKM2 inhibitor, an analog of shikonin modified based on its structure, had much higher SI for Vero cells (34.10) and HFF cells (16.16), suggesting that its cytotoxicity is lower than that of shikonin (Table 3).

**FIGURE 7:**
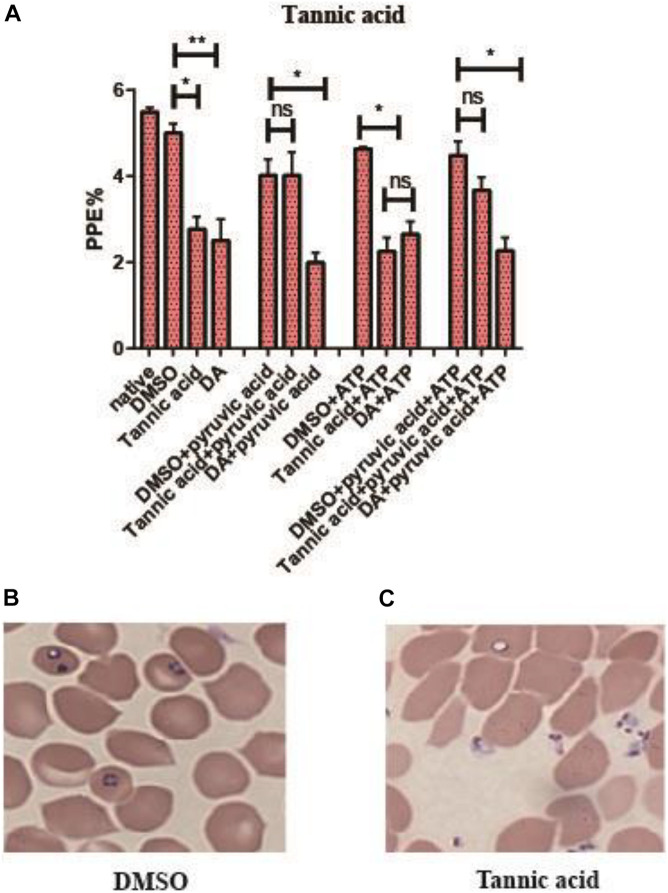
Rescue assay of tannic acid. **(A)** Statistics of parasite infection rate in the rescue assay of tannic acid. The native group represents no addition of drugs. **(B,C)** Picture of microscopy with Giemsa staining of the dimethyl sulfoxide (DMSO group) and tannic acid group. Differences in the relative growth rate of parasites were statistically analyzed using one-way ANOVA analysis, and the asterisks (*) indicate significant differences between the mixed compounds groups and the DMSO group (* represents *p* < 0.033, ** represents *p* < 0.02, and ns represents no significance). The error bar represents mean ± SD (*n* = 3), and the charts were created with GraphPad Prism 7.0.

Tannic acid could inhibit the activity of rBmPyKI and decrease the production of both pyruvate and ATP, which affected the energy metabolism of *B. microti*. The supplementation of pyruvate and ATP could provide energy to partially recover the growth of *B. microti*. According to the catalytic mechanism of PYKI, a rescue assay was performed by adding pyruvate or ATP into the culture medium. The statistical results illustrated that tannic acid (*p* = 0.03) and DA (*p* = 0.01) can significantly inhibit *B. microti* as compared with DMSO. The addition of pyruvate could rescue the inhibitory effect of tannic acid but showed no effect on that of DA, while the addition of ATP did not affect the inhibitory effect of both tannic acid and DA (*p* = 0.01). These results indicated that tannic acid could inhibit the growth of *B. microti in vitro* by decreasing the activity of PYKI. The inhibition could be rescued by the addition of pyruvate in the culture medium (Figure 7A). ATP could not partially recover the growth of *B. microti* since it cannot be transported into the cells. Interestingly, microscopy observation showed that the parasites inhibited by tannic acid were mostly located outside RBCs (Figures 7B,C). Therefore, tannic acid may reduce the possibility of parasites to invade RBCs, which can reduce the infection rate and inhibit the growth of *B. microti*.

## Discussion

Pyruvate kinase is an essential regulatory glycolytic enzyme in intracellular glucose metabolism of parasitic organisms and is a potential drug target against *P. falciparum*, *Trypanosoma*, and *Leishmania* (Ernest et al., 1994; Ernest et al., 1998; Chan and Sim, 2005). Currently, the characteristics and functions of PYKs from *Trypanosoma brucei*, *T. gondii*, *L. mexicana*, and *P. falciparum* have been well documented, but relatively little is known about the PYK from *B. microti*. Analysis of *B. microti* metabolism based on its genomic information has suggested that the lack of mitochondrial superoxide dismutase and pyruvate dehydrogenases will lead to defective mitochondrial antioxidant system. Parasites are highly dependent on glucose fermentation for energy production and redox regulation (Cornillot et al., 2012). Therefore, PYK may be a promising target for the development of new drugs against *B. microti*.

In order to evaluate the characteristics of PYK, the gene encoding BmPYKI was amplified by PCR from *B. microti* cDNA, and the recombinant protein was expressed in *E. coli*. Consistent with the data provided by NCBI, amino acid sequence analysis showed 50.20–56.12% similarity of BmPYKI to the PYKI enzymes of *E. tenella* (XP_013232798.1), *T. gondii* (XP_002364923), *P. falciparum* (XP_966251), and *T. annulata* (XP_953251), and a 43.64% similarity to human PYKI (AAA60104.1). Multiple alignments based on the amino acid sequences showed that BmPYKI shares similarities with *Homo sapiens* PKL (43.23%, BAA02515.1), PKM (43.11%, NP_001193727.1), and PKLR (43.43%, NP_000289.1). rBmPYKI had a higher affinity for PEP and a lower affinity for ADP than PYK from *T. gondii* (*K*_m_ = 0.75 mM for PEP; *K*_m_ = 0.18 mM for ADP) and significantly lower affinity for both PEP and ADP than PYK from *P. falciparum* (*K*_m_ = 0.19 mM for PEP; *K*_m_ = 0.126 mM for ADP). Moreover, the affinity for ADP is also lower than that of the corresponding enzymes from mammals (*K*_m_ = 0.35 mM). The optimal pH for rBmPYKI activity is 7.0, which is close to the blood neutral pH range and necessary for parasite survival. However, the p*K*a values of different buffer at 37°C, such as MES, PIPES, TES, HEPES, and TRIS, should be evaluated in the pH range of 5.5–8.5, to ensure pH stability during the time of enzymatic measurements. Admittedly, performing kinetic analysis not with FBP as an allosteric modulator of the rBmPYK activity is obviously a drawback of our study.

Almost all PYKs have been reported to be homotetramers, which have been well characterized, including *T. brucei*, *T. gondii*, *L. mexicana*, and *Cryptosporidium parvum* (Ernest et al., 1998; Bakszt et al., 2010; Cook et al., 2012; Naithani et al., 2015). However, there have been no major breakthroughs in anti-parasite treatment using specific inhibitors of PYKs yet. Notably, compared with apicomplexan parasites, tumor cells are characterized by high consumption of glucose. Parasites and tumor cells use the glucose of the host to produce ATP to meet the high energy demand of rapid cell growth and proliferation. According to the mechanism of glycolysis, specific inhibitors of PYK isoenzyme M2 (PKM2) have received much concern and been tested in cancer therapy. We selected 13 easily available inhibitors of PKM2 to evaluate their effect on BmPYKI activity and the growth of *B. microti*. Tannic acid exhibited a better inhibitory effect among the 13 compounds, inhibiting the activity of rBmPYKI by more than 90% at 3 μM and the growth of *B. microti* by 80% at 20 μM. Furthermore, microscopy observation revealed that tannic acid may suppress the activity of parasites to reduce their invasiveness. Shikonin, PKM2 inhibitor, and apigenin can also suppress the activity of *B. microti* through some unknown mechanisms. Therefore, it is necessary to conduct a structural analysis of BmPYKI for its characteristics, which will help to explore the mechanisms of the four inhibitors and more druggable sites for the development of selective inhibitors on *B. microti*. It should be noted that the effect of the inhibitors (shikonin, tannic acid, apigenin, and PKM2 inhibitor) on other ADP or ATP-dependent enzymes, such as hexokinase, GAPDH, and PFK-1, had not been determined before. Whether such effects can be extended to ADP or ATP-dependent enzymes and the detailed cellular mechanisms underlying these effects warrant further investigation.

In summary, a series of experiments demonstrated that tannic acid, shikonin, PKM2 inhibitor, and apigenin can inhibit the activity of rBmPYKI and the growth of *B. microti in vitro*. The biochemical experimental data may lay a foundation for the development of new inhibitors of *B. microti*. In addition, based on the cytotoxicity and high inhibitory effect, these inhibitors can be improved in structure and developed into more safe and effective structural analogs to be applied in antibabesial therapeutics.

## Data Availability Statement

The original contributions presented in the study are included in the article/supplementary material, further inquiries can be directed to the corresponding authors.

## Author Contributions

XA, LH, and JZ designed the study and wrote the draft of the manuscript. LY, SW, YA, XZ, QL, YZ, ML, XS, and FL performed the experiments and analyzed the results. All authors have read and approved the final manuscript.

## Conflict of Interest

The authors declare that the research was conducted in the absence of any commercial or financial relationships that could be construed as a potential conflict of interest.

## Publisher’s Note

All claims expressed in this article are solely those of the authors and do not necessarily represent those of their affiliated organizations, or those of the publisher, the editors and the reviewers. Any product that may be evaluated in this article, or claim that may be made by its manufacturer, is not guaranteed or endorsed by the publisher.
